# Testing big data in a big crisis: Nowcasting under Covid-19

**DOI:** 10.1016/j.ijforecast.2022.10.005

**Published:** 2023

**Authors:** Luca Barbaglia, Lorenzo Frattarolo, Luca Onorante, Filippo Maria Pericoli, Marco Ratto, Luca Tiozzo Pezzoli

**Affiliations:** European Commission - Joint Research Centre, Via E. Fermi, Ispra, 21027, Italy

**Keywords:** Bayesian model averaging, Big data, Covid-19 pandemic, Gross domestic product, Nowcasting

## Abstract

During the Covid-19 pandemic, economists have struggled to obtain reliable economic predictions, with standard models becoming outdated and their forecasting performance deteriorating rapidly. This paper presents two novelties that could be adopted by forecasting institutions in unconventional times. The first innovation is the construction of an extensive data set for macroeconomic forecasting in Europe. We collect more than a thousand time series from conventional and unconventional sources, complementing traditional macroeconomic variables with timely big data indicators and assessing their added value at nowcasting. The second novelty consists of a methodology to merge an enormous amount of non-encompassing data with a large battery of classical and more sophisticated forecasting methods in a seamlessly dynamic Bayesian framework. Specifically, we introduce an innovative “selection prior” that is used not as a way to influence model outcomes, but as a selection device among competing models. By applying this methodology to the Covid-19 crisis, we show which variables are good predictors for nowcasting gross domestic product and draw lessons for dealing with possible future crises.

## 1 Introduction

During the Covid-19 pandemic, traditional forecasting models became outdated, and their performance rapidly deteriorated. Several factors undermined their functioning. First, the Covid-19 crisis itself represented an unexpected and unprecedented shock to the world economy, and no past observations could provide a relevant signal about its potential economic impact. Second, social distancing measures imposed by governments to contain the spread of the pandemic affected both the supply and demand sides of the economy, and reduced disposable income and consumption, ultimately increasing unemployment. The uncertainty around government restrictions and policy support made it very difficult to assess their impact on national economies (see Ferrara & Sheng, 2022 and the references therein).

Despite these challenges, policymakers need short-term forecasts and nowcasts of the current state of the economy to design timely policy actions and evaluate their effectiveness in contrasting the pandemic’s adverse consequences and preserving societal wellbeing (Ferrara et al., 2022). Although they are important priorities in any policy agenda, readily available predictions are very difficult to obtain, this task being even more challenging in a period of global distress. Sharing the innovative tools and expertise developed in this experience could help other policymakers assess the economy’s real-time monitoring, providing them with a more informed and up-to-date starting point for forecast and scenario analysis.

This paper presents two major novelties that could be adopted by forecasting institutions in unconventional times. The first innovation consists in the production of a new macroeconomic data set able to consistently enlarge the standard information set at policymakers’ disposal. Many economic variables produced by statistical agencies and used by forecasters are available only at monthly (e.g., industrial production) or quarterly frequencies (e.g., national account variables, such as gross domestic product), and they are usually released with a substantial delay and subject to successive revisions. Although such macroeconomic series contain relevant and accurate information about the state of the economy, their poor timeliness might prevent them from capturing unexpected shocks during highly uncertain times. Recent studies have provided evidence of the usefulness of fast-moving measurements extracted from big data sources to complement the information of classical economic variables (see Buono et al., 2017 for a review). For example, alternative indicators, like electricity consumption (see Blonz & Williams, 2020), tone and polarity extracted from text (Algaba et al., 2023, Ashwin et al., 2021, Barbaglia et al., 2022, Thorsrud, 2020), traffic and road tolls (Askitas & Zimmermann, 2013), Google data (Aaronson et al., 2022, Choi and Varian, 2012, Ferrara and Simoni, 2022), and mobility reports (Sampi & Jooste, 2020), have proven useful to track economic activity in real time. Other studies (see, for instance, Eraslan and Götz, 2021, Lewis et al., 2020, Woloszko, 2020) instead merge some of the above alternative sources into a few factors aimed at representing the real-time reactions of the economic agents to unanticipated shocks.

In this paper, we assess the usefulness of traditional and alternative indicators to nowcast gross domestic product (GDP) in the wake of the pandemic. We complement a large amount of conventional monthly macro-series (fat data) with a set of timely high-frequency alternative indicators (big data). Among the big data variables, we include series that have already proven to be a useful proxy of economic developments, such as electricity figures, text-based sentiment indicators, and Google Trends. Moreover, among the big data variables, we add series that have not been tested in an economic nowcasting exercise. These big data sources include Airbnb review figures, air cargo and air quality statistics, measures of media attention and sentiment extracted from the Global Database of Events and Tone (GDELT) of Leetaru and Schrodt (2013), mobility indicators based on mobile phone data of Santamaria et al. (2020), and aviation figures by Iacus et al. (2020). Summing to more than a thousand variables, this makes our data set one of the biggest macroeconomic data sets to date.

In the special context of the pandemic, the selection of fast-moving indicators goes hand in hand with the use of modeling methodologies that account for the quick changes in big data variables as well as the structural relations among standard macroeconomic time series. Recent studies have shown that relying only on one model could be dangerous, since standard linear methodologies typically struggle to capture an abrupt change in economic activity (Goulet Coulombe et al., 2022, Huber et al., 2020), while more sophisticated econometric techniques might fail at accurately estimating the intensity of the recession (Carriero et al., 2020).

As a matter of fact, the second novelty of this work is a new methodology to merge an enormous amount of non-encompassing data with a large battery of classical econometric models – namely, autoregressive distributed lag models (ARDLs), mixed-data sampling (MIDAS) regression, mixed-frequency Bayesian vector autoregression (VAR), and dynamic factors models (DFMs) – with some machine learning (ML) forecasting models (such as random forests, extreme gradient boosting, stacked ensembles, and neural networks) in a seamlessly dynamic Bayesian model averaging (BMA) framework. Specifically, we introduce an innovative selection prior that is used not as a way to influence model outcomes, but as a selecting device among competing models. Following Dietrich et al. (2022), we conduct an economist’s survey in the second quarter of 2020 by asking experts about the effects of the lockdown measures on different economic activities. This allows us to set the Bayesian priors for model averaging consistently with the expected effects of government provisions implemented to stop the diffusion of Covid-19.

The advantage of using this policy information is twofold. It reduces the complexity of the nowcasting exercise by focusing only on the variables that are in line with the expected effects of policy measures. In addition, it permits a reduction to the high level of complexity, given the many different model specifications estimated. Model averaging allows us to produce the complete distribution of the nowcast, thus emphasizing the uncertainty and risk associated. The set of models and the database were dynamically expanded, making this project a particularly groundbreaking venture that Bayesian model averaging techniques could handle with a good degree of flexibility.

The empirical nowcasting assessment of GDP is performed for the four major economies in Europe, namely France, Germany, Spain, and Italy, and spans an out-of-sample period from the last quarter of 2011 to the second quarter of 2021. To separately assess our model during the Covid-19 crisis, in our nowcasting exercise we first consider data until the last quarter of 2019 and then we include pandemic observations by extending our sample until the second quarter of 2021. The results highlight the added value of the proposed forecasting strategy at both point and density forecasting during unstable times. Big data variables appear to be particularly important at nowcasting GDP in the second and third quarters of 2020 and in the first quarter of 2021. Despite their relatively minor importance in 2021, a consistent subset of big data variables is still selected among the most relevant regressors, indicating their usefulness at nowcasting.

The remainder of the paper is structured as follows. Section 2 provides an overview of nowcasting experiences during the pandemic. Section 3 describes the alternative big data used as explanatory variables and the model set. The Bayesian model averaging approach and the definition of the prior are presented in Section 4. Sections 5, 6 report on the real-time nowcasting experience during the pandemic and on the nowcast performance, respectively. Finally, Section 7 concludes.

## 2 Nowcasting the pandemic: Academia and public institutions

Various approaches have been proposed in the literature about nowcasting during the pandemic. By leveraging previous experiences in public institutions and recent academic research, we filter out data sources and modeling techniques that might fit the exceptional pandemic period and, starting from that, we develop our approach. The works of Lenza and Primiceri (2022) and Schorfheide et al. (2020) show how to handle linear VAR models as forecasting tools in the presence of extreme observations during the Covid-19 pandemic. Building on the linear settings of the two papers above, Huber et al. (2020) develop a non-linear mixed-frequency Bayesian VAR to produce monthly nowcasts of GDP using additive regression trees, and claim that they are particularly suited when forecasting extreme values, like the ones observed during the pandemic. Jardet and Meunier (2022) fit factor-augmented MIDAS to forecast world GDP using a big data set of 190 series at monthly and weekly frequencies, showing large nowcasting gains in crisis periods. The importance of weekly frequencies to track US economic activity in a timely manner is also explored by Lewis et al. (2020), who build a weekly economic index by extracting the first principal component out of 10 series, including retail sales, unemployment indexes, raw steel production, electricity output, and traffic data. This indicator is regularly updated and available on the St. Louis Fed Research website.^1^ Similarly, Eraslan and Götz (2021) extract a common factor from a set of unconventional high-frequency indicators, which also include the number of flight passengers and the pedestrian frequency in shopping districts, and construct a weekly activity index for Germany that is updated regularly and available on the Deutsche Bundesbank website.^2^ Richardson et al. (2021) focus on a much larger data set of 600 predictors to nowcast GDP in New Zealand. They show that a selected set of machine learning algorithms can outperform classic univariate forecasting methods. At the global scale, Diaz and Quiros (2020) use factor models and a set of worldwide commodity prices to extract a daily global tracker of economic activity. This index has good forecasting properties for global PMI during both normal and crisis periods.

Although the literature on nowcasting during the Covid-19 pandemic includes other relevant works (regarding works on nowcasting, we refer the reader to Babii et al., 2022, Proietti et al., 2021), we now focus on the nowcasting experience in large public institutions, namely the Organisation for Economic Co-operation and Development (OECD), the Federal Reserve Bank of New York (NY-FED), and the European Central Bank (ECB). The OECD Weekly Tracker of GDP^3^ provides a weekly nowcast of GDP growth rates, using machine learning and Google Trends data and covering OECD and G20 countries. The Tracker is one of several indicators that feed into the OECD forecast process. The forecast is computed in two steps. First, a neural network for predicting GDP growth is estimated based on Google Trends search intensities at a quarterly frequency. Second, the quarterly model’s elasticities are applied to the weekly Google Trends series to yield the nowcast. A detailed description of the methodology can be found in Woloszko (2020). In out-of-sample analysis, the model based on Google Trends outperforms an auto-regressive model that uses lags of year-on-year GDP growth. The paper also uses interpretability tools based on the Shapley value to understand the importance of different categories of searches in the forecast.

The NY-FED publishes weekly updates of US GDP estimates and other macroeconomic variables in its Nowcasting Report.^4^ The modeling approach combines Kalman filtering techniques with dynamic factor models, which allow for a parsimonious representation of the dynamics of a macroeconomic big data set. We refer the reader to Giannone et al. (2008) and Bok et al. (2018) for a complete presentation of the methods. The input of the model consists of a selected set of market-moving indicators related to the current state of the economy regarding construction, manufacturing, consumption, income, labor, and trade. Such indicators are also observed by market participants and enter the model as “news”: the weekly update considers all the data as they become available, thus replicating the real-time information flow and its impact on current economic conditions. In particular, their approach replicates the traditional forecasting process, from monitoring data releases to forming and revising expectations as data are observed. Bok et al. (2018) showed that their nowcast significantly outperforms the naive AR(1) model.

In a recent work released in the ECB Working Paper series, Cimadomo et al. (2022) propose a mixed-frequency Bayesian VAR model and claim that it matches the performance of the NY-FED nowcasting tool. This technique provides a more general structure than dynamic factors models and allows for the study of structural interactions across variables. As a consequence, an institution can easily build a narrative about the policy implications of an economic outlook, for instance via the use of standard tools like impulse response functions. Moreover, Bayesian VAR models can properly account for forecast uncertainty by targeting specific prior distribution choices. As a case study, they focus on US data that include standard macroeconomic variables, as well as on the text-based economic policy uncertainty indicators by Baker et al. (2016). In an application to nowcasting US GDP in the first quarter of 2020 during the Covid-19 pandemic, the authors show that the density associated with the nowcast of the proposed model is able to capture the economic slowdown caused by anti-contagion restrictions, while this is not the case for the NY-FED benchmark methodology.

In sum, our approach owes a lot to the literature with respect to both the modeling choice (e.g., the inclusion of non-linear models or the adoption of a Bayesian framework) and the input data (e.g., relying on a large data set with conventional and unconventional data). At the same time, our work expands the space of models and data used and provides, in comparison with existing frameworks, a flexible model selection framework that can be generalized to future crises. We hope that reporting our experience can help other researchers and practitioners.

## 3 A real-time story: Nowcasting an outlier

This section illustrates two important aspects of our work. First, we describe the extensive and heterogeneous data set that we use to capture the real-time reactions of economic agents. Then, we briefly present the forecasting models, highlighting the advantages and disadvantages of each of them.

### 3.1 The information set

During our journey in nowcasting in the wake of the pandemic, our information set grew organically. We started with a few traditional macroeconomic variables and all the alternative high-frequency data sources that we could recover. Our opinion was that an unprecedented systematic shock could not be forecasted using history but only with quickly adapting variables. Once the first official data incorporating the effect of the Covid-19 crisis became available, we also expanded the set of traditional macroeconomic indicators. The data set is composed of *fat data*, or large amounts of traditional data, and of *big data*, real-time organic information stemming as a direct sub-product of human activities. Their role in nowcasting is quite different. On the one hand, fat data are large amounts of traditional macroeconomic series. They are published by statistical offices, come with varying delays, and are important for nowcasting in normal times. On the other hand, big data are fast-moving and might provide an early signal of the reaction of economic agents to a shock. Nevertheless, big data are no panacea. They are often not a representative sample of the whole population, since they do not stem from correct statistical sampling procedures. Rather, big data are the direct product of some specific human activities. Therefore, they may only cover the activities of a population with a bias.

One of the main difficulties in the use of big data, mainly due to their novelty, is that they represent almost uncharted territory when economic forecasting is considered. In particular, the literature offers little guidance in selecting relevant big data variables. Some of the variables may provide additional predictive power toward the variable of interest. Others, although intuitively correlated, may be useless in practice because they are too noisy. Consider, as an example, the level of CO2. Our intuition suggests a correlation with the level of economic activity, but there is little literature about whether it works in practice. For instance, if we consider a sampling station located far away from productive structures, its signal may be more informative about weather and wind conditions, representing noise in a production-related perspective. Decades of econometric works have instead explored the statistical relations across traditional macroeconomic series, and one can rely on past experience to select the most important variables to use in a model. A classical example is industrial production: it is a well-documented and widely used explanatory variable at monthly frequency for GDP. Overall, traditional macroeconomic variables can be very informative for nowcasting purposes, despite their poor timeliness.

On the other hand, alternative data can provide a timely indication of the reaction of economic agents to a shock, although their signal can potentially be very noisy and biased. In order to balance the informativeness of fat data and the timeliness of alternative indicators, we build a big data set that combines different data types: (i) traditional macroeconomic indicators and survey and financial data (*fat* data), and (ii) alternative data (*big* data). The first data type consists of monthly and quarterly survey-based indicators about business and consumer sentiment, as well as official statistics and financial variables observed at daily frequency. Our selection includes and expands the financial and macroeconomic data set employed by Schumacher (2016). The second data type gathers a number of alternative data: fast-moving variables about air quality, transport, energy production and consumption, internet searches, text-based sentiment indicators, and Covid-19-specific indicators. Such variables are not necessarily related to finance or to the current state of the economy, but they can provide a timely signal of the economic agents’ reaction to the anti-contagion restrictions and their expectations about the future severity of the economic slowdown.

**Fig. 1:**
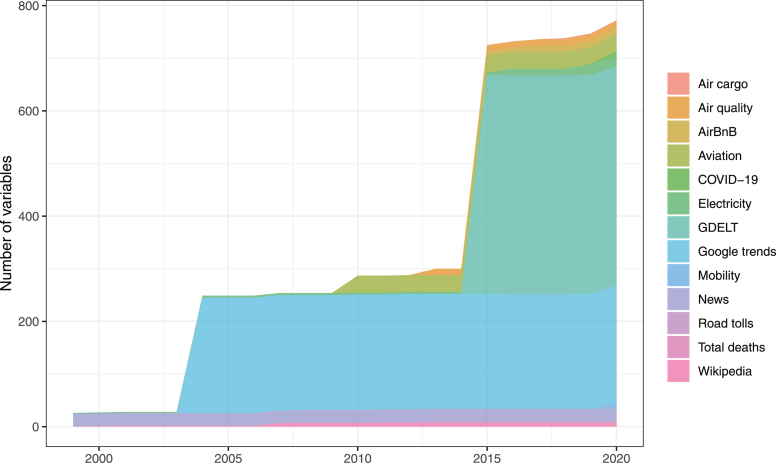
Number of big data variables available over time by variable group.

Focusing on alternative data, it is important to notice that the time span of their samples varies largely across variables. Fig. 1 reports the number of time series available in each year of analysis by variable groups. A group of approximately 20 time series starts before the 2000s, including Wikipedia searches, total deaths, road tolls, and news-based sentiment indicators (Barbaglia et al., 2021). Google Trends represent almost 200 series that become available in 2004, together with electricity consumption and production statistics. The variables about the aviation sector – namely the number of passengers and average revenues of Iacus et al. (2020) – and the air quality indicators become available in 2010 and 2013, respectively. In 2015, Airbnb review figures started alongside the hundreds of sentiment- and media attention-related measures extracted from the GDELT database (Consoli et al., 2021). Finally, in 2020, Covid-19 indicators entered the data set together with the mobility indicators of Santamaria et al. (2020).

New data enter the model as they are available, thus reproducing the real-time information flow of vintages, expectations, and revisions discussed in Bok et al. (2018). As of the outbreak of the pandemic, the data set has been updated weekly (Fridays at 2:00 p.m. CET): in each update, we collect the latest available information for each series, starting from January 1995. Data are aggregated at monthly frequency by averaging. The final data set contains 1134 series for the euro area as a whole and for the four largest European economies, namely France, Germany, Italy, and Spain, making it, to the best of our knowledge, one of the biggest data sets explored in the nowcasting literature. Online Appendix A and the tables within provide a detailed description of the data set and data transformation.^5^

### 3.2 The model set

One model never fits all, and this is especially true during the Covid-19 crisis, when existing models seem to become quickly unreliable. In the tradition of forecasting under model uncertainty (Kapetanios et al., 2008), we use many models, including well-known econometric modeling strategies (ARDL, DFM, and VAR), MIDAS, and non-linear specifications such as ML models, to produce individual forecasts. We then combine their predictions in a second stage.

• ARDLs: Autoregressive distributed lag models. These are standard, unrestricted regressions where the dependent variable (GDP) is a function of its own past and of current and past values of an explanatory variable (x). We consider a high number of models, each featuring past values of GDP and one explanatory variable. We also consider transformations of the variables as additional regressors. Therefore, in different equations, the same x variable may enter in levels, quarter-on-quarter or month-on-month growth rates, and in different lags (up to three months). Non-stationary specifications are dropped.
• DFM: The dynamic factor model has proven to be a successful reduced-form econometric model both for nowcasting and forecasting purposes. This class of model is intensively employed by central banks and international organizations for monitoring the state of the business cycle, and for computing short-term projections of macroeconomic variables such as growth and inflation. In this paper, we use the version of the model proposed by Giannone et al. (2008), which was developed to nowcast quarterly series through indicators available at a higher frequency and subject to frequent revisions. The model is estimated in two stages. In the first stage, stationary monthly indicators are employed to estimate a monthly factor model via principal components, as in Stock and Watson (2002). In the second stage, the monthly factor is aggregated at the quarterly frequency, and is employed in a bridge equation to nowcast the quarterly series of interest. In our application, the principal components are extracted from a set of 20 indicators, including the main aspects of the business cycle.
• MG-MIDAS: MIDAS estimation with big data using modal grids. MIDAS estimation handles regressors with lower frequency using temporal aggregation with a parameterized weight distribution (Ghysels et al., 2020). Once the aggregation is done, the estimation is equivalent to ordinary least squares. The proposed method exploits this feature and, given the weight function, computes a grid of weights such that each set of weights has its mode on a different lag. Then aggregation is performed for each set of weights and each regressor, resulting in a number of new aggregated regressors equal to the number of original regressors multiplied by the number of weight sets. The selection of aggregated regressors is then performed using the generalized least squares screening (GLSS) proposed in Yousuf (2018). Values of parameters of the weight function originating the most significant aggregated regressors are stored and reused as initial values in a final maximum-likelihood estimation of the MIDAS regression. This methodology allows for pre-selection among a large number of variables while maintaining contributions from a wide distribution of lags in the final estimation.
• MF-BVAR: Mixed-frequency Bayesian vector autoregression by Schorfheide and Song (2015). This approach allows us to jointly model variables observed at quarterly frequencies (e.g., GDP) with monthly ones (e.g., unemployment). Similar to Schorfheide et al. (2020), we adopt the standard setting for the Bayesian estimation of the model and select only a limited number of variables.^6^ A major benefit of such a model is that it provides point and density forecasts, while jointly modeling multiple variables observed at mixed frequencies. As it is a linear multivariate model, it does not account for complex non-linear effects or interactions among variables.
• ML: We use several machine learning models – a deep neural network (NN), stacked ensembles regression (SE), random forests (RF), and extreme gradient boosting (XGB) (refer to Hastie et al., 2009 for an introduction to the first three techniques, and to Chen & Guestrin, 2016 regarding XGB). The NN is a multi-layer network, based on randomized five-fold cross-validation for parameter tuning and on a grid search for the selection of the number and size of the hidden layers. The SE is a supervised algorithm that finds the optimal combination of a set of learners by “stacking”. The RF is a “bagging” algorithm that generates a forest of classification trees, where each of these weak learners is fitted on a random subset of rows and columns. On the other hand, the XGB is a “boosting” algorithm which fits the weak learner on sequentially re-weighted versions of the data, including two penalties on a large number of leaves and on the leaf weight of the classification tree. Although the list of machine learning models is not exhaustive, the implemented models represent the most important machine learning techniques, ranging from boosting, bagging, and penalized regression to neural networks. We feed the ML models with the full data set in real time and with a one-quarter lag, and select the best model based on a squared loss: in the majority of cases, NN is the best performing model. We repeat the procedure on 100 bootstrap samples, and obtain a point forecast for the median and an associated density.^7^

The breadth of our model set captures various aspects of the economic dynamics that might play a key role in providing an accurate nowcast. Dynamic factor models represent a well-established nowcasting tool that parsimoniously represents complex data structures. Linear mixed-frequency techniques simultaneously model monthly and quarterly frequency variables, account for their structural relations, and provide a reliable long-term view of the future economic outlook (Schorfheide et al., 2020). Machine learning models are particularly suited to work with a large number of regressors and, most importantly, are able to promptly capture non-linear dynamics in the data (Babii et al., 2022, Richardson et al., 2021).

## 4 Bayesian model averaging

### 4.1 Forecasting with BMA

Forecasting with many regressors under high model uncertainty is a challenging task. First, the presence of more than a thousand regressors makes standard econometrics unfeasible, due to the curse of dimensionality. Additionally, the practice of estimating and using a single specification ignores model uncertainty, leading to over-confident inferences. For this reason, we opt to combine the information contained in different forecasts, with non-nested models and different information sets. BMA provides a coherent mechanism to account for model uncertainty while allowing for estimations in the presence of many regressors.

BMA has been made popular in the economic literature by Sala-i Martin et al. (2004) and later used in various economic applications (e.g., Proietti & Giovannelli, 2021 rely on BMA to nowcast monthly GDP). It allows the researcher to be agnostic on the specification, estimate a large battery of models, and average them based on their forecasting accuracy. The advantages of BMA include the possibility of using parsimonious models that yield more stable estimates because of the fewer degrees of freedom that are used in individual models. Also, BMA can help identify important regressors, making the results more informative and easier to interpret. Crucially, it accounts for model uncertainty, and can be used as a tool to select the best indicators.

In this paper, BMA is used to deal with several econometric issues, including the short data span for some of the big data, the very high number of potentially relevant models, and the high risk of misspecification due to the size and noisiness of the database. It is important to note that in our case, as it is common practice when institutions use multiple models for forecasting, the models to be merged are non-nested. This raises specific problems, because Bayesian posterior odds comparisons are inconsistent when selecting among non-nested models. Hong and Preston (2012) study the case of non-nested model averaging and show that the averaging weights are non-degenerate even in large samples, as long as the models are sufficiently close to each other and none of them is the correct one. While Bayesian posterior odds and the Bayesian information criterion (BIC) are consistent for selecting among nested models, they are not consistent for selecting among non-nested models. Following Hong and Preston (2012), we resort to the non-nested information criterion (NIC), which, in large samples, selects the most parsimonious model even if the models are non-nested. We check for the robustness of the BMA by using both the BIC and the NIC. In both cases, we exclude those models for which the number of parameters cannot be determined (e.g., neural networks).

We join the high number of available models and compute BMA weights on the basis of predictive likelihood. As an out-of-sample evaluation, predictive likelihood has the advantage of being robust to different parametrization choices and degrees of freedom. To take advantage of the growing sample and allow the models to progressively adapt to the crisis, the weights $w_{i,t}$ on the BMA are updated in real time on an expanding estimation window:

(1) $$w_{i,t}=Pr\left(M_{i}|y^{1:t-1},X^{1:t-1}\right)\propto \overset{t}{\underset{\tau =1}{\prod }}Pr\left(y^{\tau }|y^{1:\tau -1},X^{1:\tau -1},M_{i}\right)Pr\left(M_{it}\right),$$

where $M_{i}$ is the set of candidate models, $y^{1:t-1}$ is the past of the endogenous variable, $y^{t}$ is the value observed at time t, $X^{1:t-1}$ denotes exogenous variables available up to time t−1, $Pr\left(M_{it}\right)$ is the prior probability of model $M_{i}$ (note that it can vary over time), and $Pr\left(M_{i}|y,X\right)$ is the posterior probability of model $M_{i}$. The weights are normalized at every t to sum to one.

An alternative, widely used technique is Bayesian model selection (BMS), where all the weight is given only to the best model at each point in time:

(2) $$w_{it}^{\prime }=I\left(\underset{i}{\mathrm{argmax}}\;w_{it}\right).$$

The model priors, $Pr\left(M_{it}\right)$, are typically assumed to be equal, or a decreasing function of model complexity when simple models are preferred. In our case, we use an equal prior for most of the sample, but in t=2020 Q2 and t=2020 Q3, we introduce our survey-based prior, detailed in the next section. BMS results are usually reported along with BMA, as the approach performs comparatively well in turbulent periods. However, BMS does not consistently beat the benchmark, because the choice of different models at each point in time often introduces “model noise”.

### 4.2 The selection prior

A distinctive feature of the process of nowcasting was the increasing, massive amount of models and data sets. Among the models, for example, some were by construction highly reactive to new information (e.g., MG-MIDAS), while others put a higher emphasis on continuity and on the covariances among variables (e.g., MF-BVAR). ML models belong to yet another category, as they emphasize non-linearities to an extreme degree, but they are difficult to interpret. To complicate matters further, each class of models uses different information, exploiting a subset of the data used. Recall that the database is not only very extensive, with hundreds of traditional macro and big data time series, but also includes hundreds of untested “big data” series: some of them may provide good trackers of economic developments during the pandemic, but most would simply add noise.

Before Covid-19, we would start BMA using an equal-weight prior and derive the final weights on the basis of past performance. But 2020 was not a normal year. When we started nowcasting, exceptional circumstances and policies were in place. In order to distinguish models that captured these exceptional circumstances from those that could not, we needed to include in our prior a selection device. For the second quarter of 2020, we decided to resort to an economists’ survey. We exploited our institutional environment, the independent assessment of economic activity and policy intervention available to us by the internal European Commission (EC) channels to shape our priors and skim the space of models. Our survey involved 40 economists and took place in April 2020.^8^ We did not ask for a forecast of GDP directly, because this would have been very difficult and subjective. Instead, we focused on the effect of the lockdown measures on different economic activities. The survey only included the following main question: “According to your opinion, and looking at the country/region where you live, what is the fraction of activity levels which is lost due to lockdown, on a scale from 0 to 100, in these sectors of the economy?”. A list of NACE sectors followed, and for each sector the answer could be chosen as follows: (i) unaffected, (ii) 25% loss, (iii) 50% loss, (iv) 75% loss, or (v) complete shutdown. Before leaving the survey, participants were asked about a self-assessment on how familiar they were with the economic situation and the country where they live, but these questions were not used in the analysis.

**Fig. 2:**
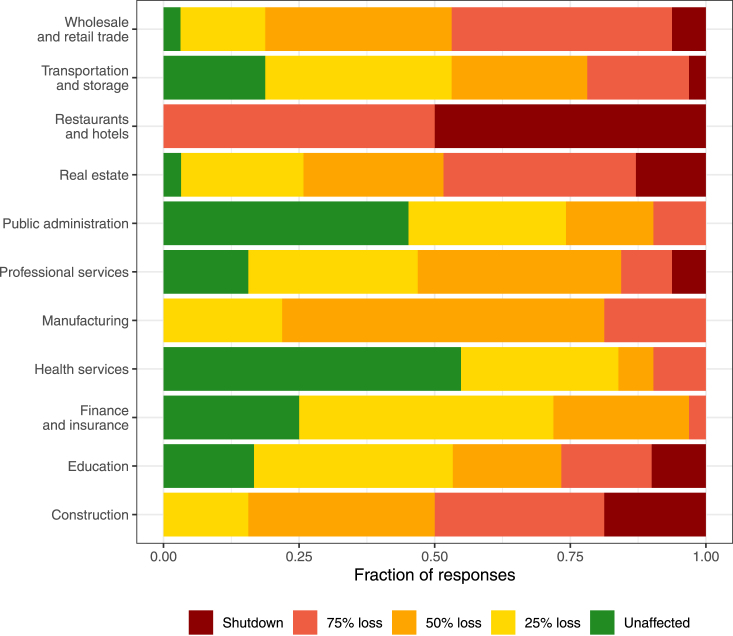
Survey of economists for 2020 Q2: answers to the question “According to your opinion, and looking at the country/region where you live, what is the fraction of activity levels which is lost due to lockdown, on a scale from 0 to 100, in these sectors of the economy?”.

Fig. 2 reports the results of the survey. Respondents agreed homogeneously that restaurants and hotels would suffer most from the restriction measures imposed by governments, with half of them suggesting that this sector would go through a complete shutdown. Other activities that would be largely affected were wholesale and retail trade, real estate, and construction, with more than half of the respondents indicating that the lockdown measures would cause at least a 75% loss. On the other hand, survey participants suggested that public administration, health services, and financial activities would be unaffected by the pandemic restrictions. From these questions, we evaluated the mean effect (common to all countries) of the lockdown as follows:

(3) $$\mu_{L}=\frac{meanClosure*daysOfClosure}{90},$$

where meanClosure indicates the average closure of the economy resulting from the survey, aggregating the sectors according to their weight in GDP; and daysOfClosure is the number of days the lockdown was in place in the quarter (assumed to be 90 days). The variance of the prior $\sigma_{L}^{2}$ is computed using the variability in single-respondent assessments. The prior associated to each model forecast $M_{it}$ is obtained as follows:

(4) $$Pr\left(M_{it}\right)\propto \left\{\begin{array}{cc} \phi \left(\frac{M_{it}-\mu_{L}}{\sigma_{L}}\right) & t\in \left(2020\;Q2,2020\;Q3\right)\\ 1 & otherwise \end{array}\right\},$$

where ϕ represents the standard normal density.

**Fig. 3:**
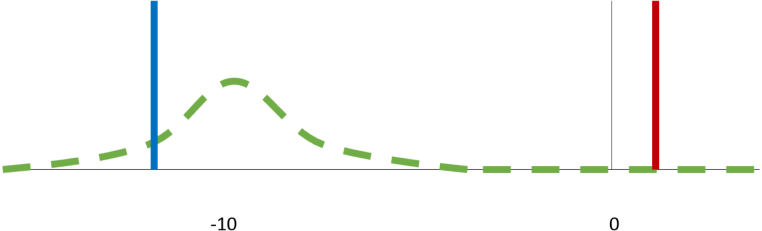
Example of the selection prior and two nowcasts.

As an example, Fig. 3 shows two models: the Red model and the Blue model. The colored bars show their point forecast in 2020 Q2, when lockdown policies led to the deepest part of the recession. The Red model includes variables that are either backward-looking (lags) or big data that do not react to the Covid-19 crisis. The Blue model, by contrast, includes a leading indicator for GDP during Covid-19. The green distribution is the prior calculated from the survey. In this case, the survey prior will lower the prior weights of the Red model in the BMA, while the prior weights of the Blue model in the BMA will be higher. The final weights of the two models in the BMA will depend, of course, on the posteriors, and it cannot be excluded that a high likelihood leads the Red model to dominate over the Blue one despite a worse prior. The estimated effect of the policies is not added to the no-policy forecast, but serves the purpose of selecting those models that react realistically to the crisis.

It should be noted that the use of survey information to twist forecasts is not new. For instance, the “democratic prior” in Wright (2013) uses the predictions of survey respondents as priors to discipline nowcasting. Here, contrary to them, we do not twist the prediction of the models, but we use the survey more as a selection device. Our “selection prior” for model averaging fulfills two distinct purposes. First, it provides information about the effects of the lockdown measures. Given that their precise impact is unknown in real time, we do not add it to the nowcast in a dogmatic additive manner, but rather as a Bayesian prior. The use of a prior to input the effect of government provisions improves on a simple additive policy measure, as it accounts for the uncertainty around the existing estimations. Second, the prior is added while averaging across all models; each model will nowcast without the prior, but some models (for example, those that turn out to be completely unresponsive) will be dropped in the model averaging step. By providing additional information about an important component of the nowcast, the prior helps downplay those models and variables that do not have predictive power during the crisis. In particular, variables that are non-reactive to the Covid-19 crisis are downplayed in model averaging.

To summarize, the weights for model averaging in the BMA were calculated by using an equal-weight prior before the pandemic. When the Covid-19 crisis hit the European economy in 2020 Q2 and lockdown policies were put in place, our prior was not equal weight anymore, but we relied on the survey as a prior for the evaluation of the predictive likelihood of the 2020 Q2 forecast. In 2020 Q3, distancing measures were abandoned and daysOfClosure dropped to zero. The prior for 2020 Q3 therefore had mean $\mu_{L}$ equal to zero while its variance (the $\sigma_{L}$) was assumed to be the same as in 2020 Q3. In the successive quarters, an equal-weight prior was restored.

The use of the survey allowed us to exploit big data in a way that avoids hand-picking, while accounting for unprecedented policy responses adopted by governments. Among the advantages of this prior, we highlight its Bayesian nature (i.e., it is drawn from a completely independent source of information) and the fact that it does not impose an exact evaluation of the policy effects. We also note that, while we see this specific survey as a valuable addition in the Covid-19 context, there is a more general scope in including surveys as selection priors^9^ but the solution is to be found on a case-by-case basis. In “normal times”, for example, the early information provided by high-frequency (e.g. monthly) surveys, such as consumer confidence or professional forecasts of GDP, could be used as a model selection device. This extension will be the object of subsequent research. Extrapolating from our promising exercise, we conjecture that using surveys as a selection device could be an efficient alternative to their use in disciplining other forecasts.

In summary, the forecast is produced in two steps. First, at each time t, we estimate the individual models and produce the associated forecasts. Second, we use a prior with equal weights for each type of model, with the exception of the policy prior used during the first lockdown, and we update it with the (predictive) likelihood to compute the final weights of the BMA. In line with the Bayesian concept of inclusion probabilities, we also assess the importance of the variables by attributing the weight of each model to the variables that appear in it.

## 5 Real-time forecasting during the pandemic

The idea of having a two-step process for nowcasting, including several non-nested models and a model averaging step, came from theoretical and institutional considerations. Bayesian econometrics has shown that model averaging hedges against major mistakes, and often performs better than most models of the averaged pool. Therefore, this seemed to be a good approach in a time of increased uncertainty. On the institutional side, the many different non-nested models available at the EC Joint Research Centre were natural candidates as starting points, and BMA provided a natural framework for their joint use and assessment. This approach proved to be remarkably resilient and flexible over time.

In the first months of 2020 Q2, it became clear that the existing models were mostly missing the upcoming downturn. This led to additional research in two directions. On the one hand, we increased the space and type of models used in our forecasting applications. On the other hand, we performed a large-scale search for possible real-time indicators, which immediately led to the use of organic big data information. The first stream of expansion led to the use of mixed-frequency models (MF-BVAR and MIDAS) and non-linear frameworks (NN and other ML techniques) aiming at capturing extreme events. The second stream leveraged the wide range of research interests and data production at the EC Joint Research Centre and the help of EC Directorate General for Economic and Financial Affairs. As a result of this collaboration, we collected more than 1000 time series.^10^

When nowcasting for the third quarter of 2020, the opposite problem arose. In the months between July and September 2020, the confinement measures were removed in most places, so any plausible model would need to include a bounce-back of GDP. Among the models adopted, both with traditional and big data, there were two prominent behaviors. On the one hand, models with an important autoregressive component tended back to baseline, but at a reduced speed. On the other hand, other models extrapolating on the estimated non-linearities (i.e., mostly ML models) would suggest an even further deterioration of the economic situation. Besides, linear or semi-linear models with big data would go in all possible directions, depending on the information set. For example, models including information on flight transportation or tourism – which remained subdued, due to remaining constraints on international movement and consumer choices – would still indicate economic degradation. On the opposite side, models considering information from industry, a sector that rebounded quickly, would suggest a prompt recovery.

This gave us the opportunity to further streamline the models used, by imposing the additional prior on 2020 Q3 that the measures would not apply, thereby lowering the prior weight of models very far away from a level recovery. From 2020 Q4 onward, the equal-weight prior was used to further re-weight the models. The BMA structure seamlessly accommodated this evolution.

### 5.1 Best variables

The breadth of the information set plays a key role alongside the flexible modeling strategy. After the release of each quarterly GDP, the BMA reassesses the pool of models and produces posterior probabilities (so-called inclusion probabilities of the BMA) for each explanatory variable. In this section, we use these posterior probabilities to identify and report the most important regressors in each quarter.^11^

**Fig. 4:**
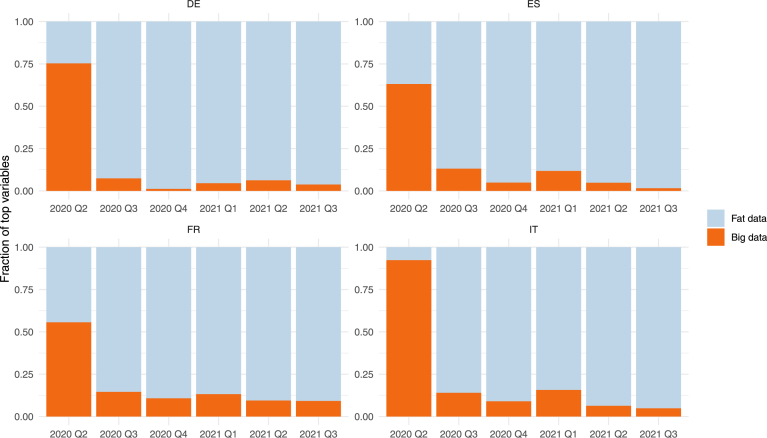
Best variables selected by BMA: Fraction of *big* data and *fat* data in each quarter.

Fig. 4 shows the proportion of *fat* and *big* data, as detailed in Section 3, among the best variables selected by the BMA modeling strategy when summing the variable contribution across all months within a quarter. Our real-time forecasting exercise started in the second quarter of 2020, when the first nowcasts were produced. In this period, big data played a key role, as they could provide timely early-warning signals of the economic deterioration that the anti-pandemic restrictions were imposing in the European economy. Interestingly, the relevance of big data varied considerably across countries. For example, approximately 80% of the best variables in Germany and Italy belonged to the big data group, compared to 60% in France and Spain. Starting from 2020 Q3, the proportion of big data dropped, ranging between 10% and 15% in all countries. As a matter of fact, big data provided timely signals of economic deterioration during the outbreak of the pandemic when severe restrictive measures were implemented to contain the spread of the virus. When the state of emergency reduced and lockdown policies were progressively lifted, traditional macroeconomic and financial indicators largely replaced the alternative data sources. The drastic drop in the fraction of big data among the best variables has also to be imputed to an important increase in the size of the information set. Indeed, in the last quarter of 2020, we added approximately 60 new variables from Schumacher (2016), thus artificially increasing the proportion of fat data. It is worth noting that big data again gained relative importance in the first quarter of 2021, when uncertainty about the efficacy of vaccine campaigns and the spread of new Covid-19 variants again undermined the European economic outlook. Despite the relatively minor importance of big data after 2020 Q2, a consistent subset of alternative indicators was selected among the best variables.

**Fig. 5:**
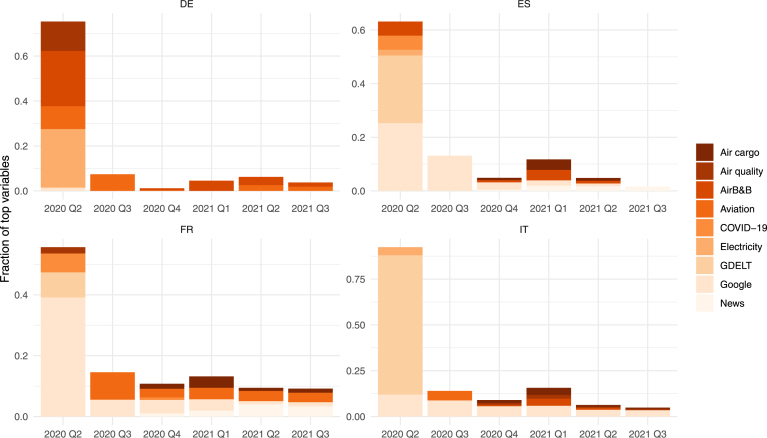
Best variables selected by BMA: focus on *big* data.

Fig. 5 reports the detailed proportion of best variables taken from the big data presented in Table A.4 in the online appendix. In the first quarter of the analysis, we observe that a number of fast-moving variables provide an early signal of the unexpected economic degradation caused by the pandemic. Electricity represents approximately 25% of the best variables in Germany, with consistent proportions in Italy and Spain. The early days of the lockdown saw an abrupt change in the consumption and production patterns of households and firms, which caused an unprecedented reduction of any economic activity. Air quality indicators report a slowdown in the industrial and transportation sectors through a reduction in the level of pollution and are selected in Germany, while Airbnb occupation figures are picked out in Germany, Spain, and Italy, representing the limited travel possibilities. The GDELT indicators that measure media attention and sentiment provide a very useful signal for Italy, where they represent approximately three-quarters of the best variables. Starting from the third quarter of 2020, the proportion of big data stabilizes and some variables seem to be consistently selected. Aviation figures from Iacus et al. (2020) are present in all successive quarters in Germany and France. The text-based sentiment indicators about the current state of economic activity by Barbaglia et al. (2021) are selected in France and Spain, while Google Trends and air cargo figures are among the best variables in France, Spain, and Italy.

Taking a deeper look at the big data variables that make the most important contribution to GDP nowcasting during the pandemic, we can observe some common patterns. First, the variables that are selected among the best ones are timely, meaning that they are published with no delay and provide a swift signal of the unexpected shock caused by the pandemic. They are not necessarily high-frequency, as among the best variables we find daily (e.g., electricity or news indicators), weekly (e.g., air cargo), and monthly (e.g., Google Trends or aviation) indicators. This suggests that the frequency of publication is not a key feature, as long as the variables are available with no delay and provide a clear signal. Second, the variables need to have a long-enough time series. For instance, no indicator of social mobility or Covid-19 official statistics (confirmed cases, deaths, and recoveries) is selected among the best variables. Even though they provide a timely and high-frequency signal of the pandemic development, their time series length is too short to bring additional information to the forecasting models. Third, if we look at the big data indicators of news and media coverage in our sample (i.e., GDELT, Google Trends, and news), we observe that certain topics are more prominent than others. Among the Google Trends that are selected as the best variables, there is a clear prevalence of job-related topics. For instance, internet searches about unemployment, social security, and jobs (e.g., “curriculum vitae” or “motivation letter”) appear to be among the best variables in all countries. The importance of job-related topics is also confirmed when looking at which GDELT indicators are selected, where media attention on labor and macroeconomic issues seem to be a relevant predictor for GDP. As for the text-based indicators from news, the most important indicator is the one collecting a general sentiment about the state of the economy, while other indicators about inflation, financial markets, or industrial production are not selected.

While the results in terms of the most important predictors are inherently linked to the specific economic conditions imposed by the pandemic, we can generalize a few insights about nowcasting with big data that might be useful in future crises. First, having access to a large number of regressors is a very relevant feature, as no single indicator proves to be the best regressor across countries and quarters. Then, the variables need to have a long-enough time series to be included in the forecasting models. Finally, it is important to include fast-moving big data variables that provide information about areas of the economic activity linked to official statistics (e.g., media attention about unemployment), since their timely signal can anticipate the future outcome of the official figure that will be published with a delay.

## 6 Out-of-sample nowcast assessment

This section provides an out-of-sample assessment of the nowcasting performance of the proposed BMA model for the four countries in the analysis. We produce monthly GDP out-of-sample nowcasts from the last quarter of 2011, using an expanding window setting, which we prefer over a rolling setup, given the short time length of the quarterly dependent variable. To separately assess our model during the Covid-19 crisis, we first run our nowcasting exercise considering data until the last quarter of 2019, and then we include pandemic observations by extending our sample until the second quarter of 2021. The nowcasts of the proposed model are assessed against two benchmarks, namely an autoregressive (AR) process of order 1, and a random walk (RW). Notice that, for each quarter, we report the nowcast performance of the BMA at the end of each month included in the quarter. On the other hand, for the RW and AR(1), we obtain one unique nowcast for each quarter. We evaluate the model performance in the terms of both point accuracy and nowcast density.^12^

### 6.1 Point nowcasting

We assess the point accuracy of the BMA model in terms of the mean absolute forecast error (MAFE).^13^ Let $y_{t}$ be the actual GDP values at time t, and let ${\overset{ˆ}{y}}_{t}^{h}$ be the out-of-sample nowcasted GDP values for the proposed model at horizon h, with 1≤h≤3. We then define $\epsilon_{t}^{h}={\overset{ˆ}{y}}_{t}^{h}-y_{t}$ as the out-of-sample nowcast errors. Notice that h refers to three different sets of nowcasts. More precisely, the first set refers to the nowcast that is made in the first month of the quarter, that is, January, April, July, and October. The second set considers the nowcast that is made in the second month of the quarter, that is, February, May, August, and November. The third set considers the nowcast that is made in the third month of the quarter, that is, March, June, September, and December. Our MAFE metric is as follows:

(5) $$MAFE_{h}=\frac{1}{T^{*}}\overset{T^{*}}{\underset{t=1}{\sum }}|\epsilon_{t}^{h}|,$$

where $T^{*}$ is the total number of nowcasts produced, namely 33 nowcasts when we consider the out-of-sample period from 2011 Q4 to 2019 Q4, and 39 nowcasts when we include the pandemic observations until 2021 Q2.

We report the results relative to the MAFE of the RW: a value smaller than unity indicates better performance than the benchmark. Notice that the results are shown across different horizons to analyze the performance of the proposed model relative to the RW as we approach the release date. We also calculated the corrected version of the (Diebold & Mariano, 1995) test proposed by Harvey et al. (1997) to check whether the performance of the BMA model is significantly better than the RW benchmark. The null hypothesis is that the two models have equal predictive accuracy, while the alternative is that the BMA model has higher predictive accuracy than the benchmark.^14^

The upper part of Table 1 reports the median point nowcast relative to the RW for the AR(1) and BMA models in the three nested months when excluding the Covid-19 crisis period from the time sample. In all countries, the BMA outperforms the RW benchmark as well as the AR(1). As expected, the nowcast performance of the BMA improves as time passes. Its relative MAFE decreases over the months within each quarter, correctly representing the information flow (Bok et al., 2018): the closer to the end of the quarter, the easier it is to make a nowcast. The performance gains obtained by BMA are always visible when considering the nowcast in the second and third months of the quarter, where we observe relative gains of approximately four times that of the benchmark. In Germany, the BMA nowcasts are significantly more accurate than the RW ones in all three months in the quarter, while in France and Italy, only the second and third months are to be preferred over the benchmark. On the other hand, in Spain, we observe no statistically significant difference among the nowcasts.

If we include the Covid-19 crisis in the time sample, the results confirm the figures discussed above. The lower part of Table 1 reports the point nowcast performance considering the expanding window until the end of 2021. In all countries, the relative gains over the benchmark become greater than in the pre-Covid-19 period. In particular, the German BMA nowcasts are significantly different from the RW ones at 5% significance in all three months within the quarter. In the remaining countries, the significance is limited to the second and third months, and it is at the 10% confidence level.

**Fig. 6:**
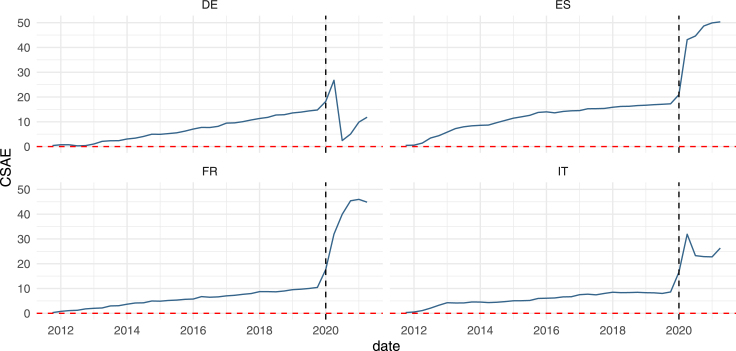
Relative difference in cumulative sum of absolute errors of the proposed model with big and fat data with respect to a model with only fat data: if above zero, then the proposed model performs better. The vertical line corresponds to 2020 Q1.

What is the added value of big data relative to fat data? We consider a BMA model that contains only fat data as a benchmark to be compared with the proposed BMA model containing both big and fat data. In Fig. 6 we plot the difference in cumulative sum of absolute errors (CSAE) between these two models. Positive values indicate that the model with big and fat data performs better than the benchmark model with only fat data (Goyal & Welch, 2003). Until 2019, the proposed model consistently performs better than the benchmark, and it accumulates a gain of approximately 10–15 percentage points in all countries. With the advent of the Covid-19 pandemic, the relative gains of big data increase swiftly, with an upward spike in CSAE across all countries. In Spain and France, the added value of big data is very pronounced and grows monotonically across all quarters of 2020; it stabilizes in 2021. On the other hand, in Germany, and to a lesser extent also in Italy, the BMA model with big data seems to provide added value only in the first two quarters of 2020, while its performance deteriorates in the remaining quarters of that year. In 2021, big data are again relevant, reversing the negative trend of the latter part of the previous year. Overall, the evidence in Fig. 6 highlights the added value of big data relative to fat data across the whole out-of-sample period. That is, the proposed model attains a lower CSAE than the benchmark at the end of the time sample, with big data delivering the most pronounced gains in the first months of the pandemic.^15^

**Table 1:** Out-of-sample point forecast model evaluation in terms of MAFE relative to a random walk model. We also show the corrected version of Diebold and Mariano (1995) test statistics proposed by Harvey et al. (1997) for equal predictive accuracy (under absolute loss function). Here,⁎⁎⁎,⁎⁎, and⁎ denote a Diebold–Mariano significance at 1%, 5%, and 10%, respectively. The evaluation period is from 2011 Q4 to 2019 Q4 (top) and 2011 Q4 to 2021 Q4 (bottom).

	France	Germany	Italy	Spain
Pre-Covid-19				

*AR(1)*	0.87⁎⁎⁎	0.70⁎⁎⁎	1.00	1.00
*BMA - 1st month*	0.84	0.72⁎⁎⁎	1.29	1.67
*BMA - 2nd month*	0.29⁎⁎⁎	0.19⁎⁎⁎	0.56⁎⁎	0.61
*BMA - 3rd month*	0.29⁎⁎⁎	0.14⁎⁎⁎	0.48⁎⁎⁎	0.56

Covid-19 Included				

*AR(1)*	0.99	0.73⁎⁎	0.89	1.07
*BMA - 1st month*	0.44	0.54⁎⁎	0.36	0.36
*BMA - 2nd month*	0.17⁎	0.20⁎⁎⁎	0.26⁎	0.19⁎
*BMA - 3rd month*	0.10⁎	0.18⁎⁎⁎	0.34⁎	0.19⁎

*** 1% significance.

** 5% significance.

* 10% significance.

### 6.2 Density nowcasting

We evaluate the entire nowcast distribution performance of our BMA model against a bootstrapped RW density by considering percentile scoring (see Hong et al., 2016 for more details).^16^ For each time period t and set of nowcasts h, we construct nowcast errors for all the percentiles of the nowcasting density, namely $\epsilon_{q_{i},t}^{h}={\overset{ˆ}{y}}_{q_{i},t}^{h}-y_{t}^{h}$, where ${\overset{ˆ}{y}}_{q_{i},t}^{h}$ is the GDP nowcast at percentile $q_{i}$, with i=1,2,…,99. For each percentile, we obtain a loss score by evaluating the associated nowcast error through the pinball loss function.^17^ The scores are then averaged across all percentiles for all time periods to assess the full nowcast distribution. We report the density predictive score results relative to those of the RW: a value smaller than unity indicates better performance than the benchmark. We also generate bootstrapped densities for the AR(1), following the procedure of Thombs and Schucany (1990). As for the point nowcast evaluation, the results are shown across different horizons to analyze the performance of the BMA model relative to the RW as we approach the release date.

Table 2 reports the assessment using the pinball loss score. Regardless of the period considered, the performance of the BMA model improves as we approach the release date. Thus, as noticed for the point nowcast assessment, the information flow is correctly represented. Regarding the pre-Covid-19 period, in all countries we observe some relative gains with respect to the RW and AR(1), with the only exception being Spain. These improvements are mostly related to the second and third months of the quarter. In the first month, only the BMA model for Germany outperforms the benchmark, but it performs similarly to the AR(1).

These enhancements become greater for all the countries and all months in the quarter when we include the pandemic period in our out-of-sample exercise. The BMA definitively becomes the better model when compared to the RW, as well as the relative performance of the AR(1). Generally, the relative gains of our model with respect to an RW across countries and horizons are approximately two times greater than the ones obtained in normal times. We notice that, when including the Covid-19 crisis for France, the relative gains reach around three times those obtained when the pandemic period is excluded from the analysis.

**Table 2:** Out-of-sample density forecast model evaluation in terms of pinball loss metrics relative to a random walk model. The evaluation period is from 2011 Q4 to 2019 Q4 (top) and 2011 Q4 to 2021 Q4 (bottom).

	France	Germany	Italy	Spain
Pre-Covid-19				

*AR(1)*	1.00	0.79	1.02	1.00
*BMA - 1st month*	1.00	0.79	1.07	1.88
*BMA - 2nd month*	0.85	0.70	0.98	1.72
*BMA - 3rd month*	0.85	0.67	0.93	1.68

Covid-19 Included				

*AR(1)*	1.08	0.77	0.95	1.07
*BMA - 1st month*	0.63	0.65	0.52	0.57
*BMA - 2nd month*	0.35	0.52	0.51	0.48
*BMA - 3rd month*	0.30	0.50	0.53	0.48

## 7 Conclusions

Economic forecasting under Covid-19 was a challenging task, due to the high uncertainty of the development of the pandemic and the need to provide accurate figures to policymakers. While documenting the experience of nowcasting GDP during the Covid-19 pandemic at the European Commission’s Joint Research Centre, this paper proposed two major novelties. First, we studied a novel data set of more than a thousand variables taken from traditional and big data sources. Second, we forecasted GDP based on a Bayesian model averaging (BMA) framework with an innovative “selection prior”.

Our results showed the importance of timely information brought by big data to forecast a fast-moving economic environment. Overall, the BMA aggregation of the forecasts from our heterogeneous pool of traditional and machine learning models outperformed the standard random walk and autoregressive benchmarks. Moreover, the extent of these improvements in forecast accuracy increased when we included the pandemic period in our exercise. Specifically, during the pre-Covid-19 period, when evaluating the whole nowcasting distribution, the results favored the BMA in the second and third months of the quarter for the majority of countries in the analysis. The pandemic period inclusion extended the BMA advantage to all horizons and all countries. We reported, in some cases, a threefold increase in performance compared to the accuracy in the pre-pandemic sample.

Our main conclusions are the following. First, the Covid-19 period emphasizes to an extreme point two aspects that forecasters have known for a long time: no single model should be trusted, and models need to be adapted and changed over time. A two-step process based on developing different models and using model averaging proved to be the key success for this real-time nowcasting exercise in our institution.

Second, in periods of abrupt change, it is crucial to enlarge, update, and adapt the information set as much as possible. Timely big data signals were found to be decisive during the pandemic, but additional information sources were needed to filter out any noise component. In our case, information about lockdown policies in the form of a prior was crucial to increase the signal-to-noise ratio of the big data. However, our experience also showed that far from the pandemic outbreak, traditional data become more relevant and already include the response to pandemic shocks. Looking ahead, a solution to ignore the outliers, as in Lenza and Primiceri (2022), becomes increasingly realistic.

Finally, uncertainty is a key component of the nowcast and should always be communicated. Our results showed that the forecasts performed particularly well in terms of densities. This was due both to the improvement in the mean given by the big data and to the correct evaluation of the (abnormally high) uncertainty surrounding the nowcasts.

## Declaration of competing interest

The authors declare that they have no known competing financial interests or personal relationships that could have appeared to influence the work reported in this paper.

## Appendix A Supplementary data

MMC S1 .

## Footnotes

6 The input of the MF-BVAR comprises macroeconomic variables of interest (i.e., GDP, unemployment rates, and CPI), the business and consumer confidence indicators, the PMI activity indicators, and a sentiment measure about the overall state of the economy.

7 For further details on hyperparameter tuning, see http://docs.h2o.ai/h2o/latest-stable/h2o-docs/automl.html.

## Footnotes

1 Available at https://fred.stlouisfed.org/series/WEI.

2 https://www.bundesbank.de/en/statistics/economic-activity-and-prices/weekly-activity-index/weekly-activity-index-for-the-german-economy-833976.

3 Available at http://www.oecd.org/economy/weekly-tracker-of-gdp-growth/.

4 The Nowcasting Report is available at https://www.newyorkfed.org/research/policy/nowcast.html.

5 The data set is published at https://data.jrc.ec.europa.eu/collection/id-00373. Some variables could not be published, due to restrictions in data publication. The data set is regularly updated, and past vintages are available.

8 More precisely, the survey was opened on April 14, the 20th answer was recorded in the system on April 21, and the survey was closed on April 30 in order to ensure that the respondents could not use real-time information from April.

9 We refer to the use of information as a selection prior for different models, not as an additional prior or as providing information to tilt the forecast itself. The use of surveys as additional models or for tilting model-based forecasts is already widely used in the literature. For a recent contribution and literature review, see, for instance, the work of Banbura et al. (2021).

10 Online Appendix A provides a summary of the data used, with additional detail about the pre-processing requested by each model and how to deal with the “ragged edge” problem (Wallis, 1986).

11 BMA obtains the posterior inclusion probability of a candidate regressor by summing the posterior model probabilities across those models that include the variable, thus providing an indication of which regressors are most important.

12 For ease of exposition, we do not report single model performances. Indeed, this evaluation is already incorporated in the second step of the BMA approach. We report the evolution of the predictive likelihood attached to each model in the out-of-sample period in Appendix B.

13 We checked for the robustness of the results to the choice of the performance metric by also looking at the root mean squared forecast error and the mean absolute percentage error, obtaining similar conclusions.

14 Notice that our out-of-sample exercise is performed in pseudo-real time and we use the last available revised GDP data as July 25, 2022.

15 As robustness checks, we compared the forecast performance of the proposed method against two additional benchmarks, namely a BMA model without the policy prior, thus assigning equal prior weights across models, and equally weighted average model forecasts in the same spirit as (Stock & Watson, 2004). The proposed BMA performed significantly better than these additional benchmarks, thus confirming the added value of the policy prior. Detailed results are available upon request.

16 We performed robustness checks and used other measures for the density nowcast assessment, such as the continuous ranked probability score (CRPS) of Matheson and Winkler (1976). The results were similar to those reported in the paper and are available upon request.

17 See Yu et al. (2018) for a presentation of the pinball loss function.
