# Supplementary material for: Testing big data in a big crisis: Nowcasting under Covid-19
Source: Int J Forecast. 2023 Oct-Dec;39(4):1548–63. doi: 10.1016/j.ijforecast.2022.10.005 (PMC9633630; doi:10.1016/j.ijforecast.2022.10.005)
Supplement: MMC S1 — . [file mmc1.pdf]

Online appendix for

Testing big data in a big crisis: Nowcasting under COVID-19

by L. Barbaglia, L. Frattarolo, L. Onorante, F. M. Pericoli, M. Ratto, L. Tiozzo Pezzoli

Appendix A. Data description

This section provides additional details about the data set and detailed information on the transformation applied for each variable. We select data starting in January 1995 until the most recent release available. Our data set is updated with the most recently available information every week and the variables observed at weekly or daily frequencies are aggregated by taking the monthly averages. Most data are publicly available, few data series are confidential and were provided by internal sources of the European Commission[1].

Table A.1 reports the variables included as additional regressors in our models falling under the “fat” data category. We consider stock and volatility indexes to proxy the present state of financial markets. We crawl the complete DBnomics[2] data sets to extract, at monthly frequency, all financial and macro-economic variables related to the countries under analysis. Moreover, we include the complete list of variables described in Schumacher (2016).

Table A.2 describes all the regressors defined as “big” data, namely variables extracted from alternative sources that are non commonly used in economic forecasting (e.g., air quality, mobility and news indicators among others). This type of data has three main advantages: (i) they are commonly observed at a higher frequency (e.g., daily) than standard official economic statistics, (ii) they are released in real-time, with short or no publication delay and no later revision, (iii) they may provide early warnings when a rapid deterioration of economic conditions occurs. However, the signal extracted from these alternative data sources is often noisy and its relevance for forecasting purposes is harder to evaluate. Furthermore, alternative data are available with separate starting dates, raising doubts on how to properly compare different models across time points.

The majority of the big data variables in Table A.2 are publicly available and collected from published sources: for instance, aviation figures are collected from Iacus et al. (2020), mobility information based on mobile phone data come from Santamaria et al. (2020) or text-based sentiment indicators are downloaded from Barbaglia et al. (2021). Among all the variables listed in Table A.2, the GDELT indicators and Google trends are the only big data that are a novel addition to the final data set. From GDELT we extract media attention, sentiment and emotion indicators belonging to five main topics: macroeconomics and structural policies, economic growth, social protection and labour, macroeconomic vulnerability and debt, and disease. The GDELT platform[3] collects real-time news stories worldwide and,

[1] The data set is published at `https://data.jrc.ec.europa.eu/collection/id-00373`. Some variables could not be published due to restrictions in data publication. The data set is regularly updated, with the possibility to access past vintages.

[2] DBnomics available at `https://db.nomics.world/`.

[3] GDELT platform available at `https://www.gdeltproject.org/`.

Variable	Frequency	Start	Description
CDS	daily	2007-01	Credit Default Swaps at country and global level from Datastream https://www.refinitiv.com/en/products/datastream-macroeconomic-analysis
Confidence	monthly	1995-01	Sentiment indicators seasonally adjusted about construction, economic, industrial, retail, consumer and service confidence. Source Eurostat
DBnomics	daily	1995-01	Financial and macro-economic variables selected by crawling https://db.nomics.world/
Employment	quarterly	1995 Q1	Active people (from 15-64 years) seasonal adjusted, not calendar adjusted, hour worked and people seeking for a job. Source Eurostat https://ec.europa.eu/eurostat
PMI	monthly	1996-01	Purchasing Managers' Index indicators about composite output, business activity, output, orders and employment (source EC internal)
Schumacher	monthly	1995-01	Financial and macro-economic variables from Schumacher (2016)
Sotck	daily	2001-01	Stock market indexes from Bloomberg www.bloomberg.com
Volatility	daily	2000-01	Global market realized volatility from Bloomberg www.bloomberg.com

Table A.1: Independent variables: fat data.

by using state-of-the-art natural language processing techniques (see Leetaru and Schrodt, 2013), extracts themes according to popular domain expert topical taxonomies and retrieves sentiments and emotions from news. From this vast amount of data, we select only the narratives from newspapers belonging to the four countries of interest and focus on articles having at least two keywords related to each specified theme. We have used the World Bank Topical Taxonomy to understand the primary focus (topic) of each article and select the relevant narratives. From this subset of news, we construct three different sets of indicators. The first one captures media attention through the five topics mentioned above (news volume). For each country and for each topic, our measure is the count of the total number of stories focusing on each specific theme normalized by the overall number of stories published in a country. The second set of indicators provides the tonality measures of the selected news calculated using a generalist GDELT built-in dictionary and three dimensions of the Loughran and McDonald (2011) dictionary: positive, negative and uncertainty. We normalize these metrics by the overall number of stories published in a country and the number of news related to the topics of interest. The last set of indicators includes the emotional connotation of the selected narratives. We collect the word count of emotions belonging to two dictionaries, namely the Regressive Imagery dictionary of Martindale (1987) and the WordNet Affect of Strapparava and Valitutti (2004). From the first dictionary we consider only its anxiety dimension, while from the second one we select the following dimensions: anger, contempt,

disgust, fear, happiness, sadness and surprise. We also retrieve the happiness score proposed by Dodds et al. (2014). All the emotional measures are normalized by the overall number of stories published in a country and the number of news that are associated with the topics of interest.

With respect to Google Trends, we download language-specific queries about automotive market, holidays, job market conditions and teleworking. For France, we look for the following keywords: "adecco, agence emploi, annulation assurance, assistance sociale, assurance chômage, assurance voyage, auto1, autoscout, autoscout24, bon coin voiture, bureau emploi, cadremploi, chomage partiel, chomage technique, chomage, curriculum vitae, curriculum, cv, doccasion, glassdoor, indeed, indemnisation, job, jobijoba, keljob, la centrale, lettre de motivation, linkedin, manpower, mastercard assurance, mercedes benz, modèle de cv, monster, offre demploi, pole emploi, pole-emploi, poste vacant, récession, randstad, recession, recrut, resume, salon emploi, stepstone, télétravail, travail, unemployment, vacances annulation, visa assurance, voiture occasion". For Germany, we look for the following keywords: "aaa auto, adecco, arbeitsagentur, arbeitslos, arbeitslosengeld, arbeitslosenversicherung, arbeitslosigkeit, auto1, autobazar, autoscout, bewerbung, curriculum vitae, curriculum, glassdoor, hotelstornierung, indeed, job, jobbörse, jobs.de, jobware, jobworld, kurzarbeiter, kurzarbeitergeld, linkedin, manpower, mercedes benz, motivationsschreiben, randstad, recession, reiserücktrittsversicherung, reiseversicherung, resume, rezession, soziale unterstützung, stellenanzeigen, stepstone, telearbeit, unemployment, urlaubsstornierung, vita". For Italy, we look for the following keywords: "aaa auto, adecco, aiuti per disoccupati, annunci lavoro, aspi, assicurazione annullamento, auto usate, auto1, autoscout, autoscout24, bancalavoro, careerjet, cassa integrazione, cercalavoro, cliccalavoro, curriculum da compilare, curriculum vitae, curriculum, cv, disoccupati, disoccupazione, domanda di disoccupazione, domanda di lavoro, fiera del lavoro, gi group, glassdoor, indeed, indennità di disoccupazione, infojobs, inps disoccupazione, inps, job, lavori, lavoro subito.it, lavoro, lettera di presentazione, linkedin, manpower, mercedes benz, mini aspi, monster, naspi, offerta di lavoro, randstad, recession, recessione, resume, seconda mano, telelavoro, trabajo, ufficio di lavoro, unemployment". For Spain, we look for the following keywords: "aaa auto, adecco, aplicacion trabajo, asistencia social, auto1, autocasion, autoscout, carta de motivación, cotizacion, curriculum vitae, curriculum, cv, desempleo, desocupado, empleo, erte, feria de trabajo, gi group, glassdoor, indeed, infoempleo, infojobs, job, linkedin, manpower, mercedes benz, milanuncios empleo, modelos curriculum, monster, ofertas de empleo, oficina de trabajo, parado, parados, paro, plantilla curriculum, prestaciones, randstad, recesión, recession, resume, segunda mano, seguro cancelación, seguro de viaje, stepstone, subsidios, teletrabajo, teletrabajo, trabajo, unemployment, vita".

Model-specific transformations

In our application, we rely on BMA to produce the final forecasts based on the individual predictions provided by our set of models. Each of the underlying models requires specific treatment on the input and deals with the issues associated with imperfect data structures (e.g., times series with different start dates, missing values or "ragged edge" as in Wallis, 1986) in different ways. As each of the models deals with input data differently, we decided not to apply any transformation to the data set, except for the ones suggested in the original work. For instance, the text-based indicators by Barbaglia et al. (2021) are standardized to

Variable	Frequency	Start	Description
AirB&B	daily	2015-01	AirB&B number of reviews daily, last 14 days and one-day ahead forecast (source EC internal)
Air cargo	weekly	2018-01	Cargo flown in m^3 and tons (source EC internal)
Air quality	daily	2013-01	PM10, PM2.5, CO and NO2 indicators from the European Environment Agency
Aviation	monthly	2010-01	Number of passengers and average revenues from Iacus et al. (2020)
COVID-19	daily	2020-01	COVID-19 confirmed cases, deaths and recovered form John Hopkings repository `https://github.com/CSSEGISandData/COVID-19`
Electricity	daily	2016-01	Price and volume electricity consumption (corrected by weather conditions) and energy production (source EC internal)
GDELT	daily	2015-03	Sentiment indicators (tone, volume and emotions) about macroeconomy, economic growth, labour market and diseases as in Consoli et al. (2021)
Google trends	monthly	2004-01	Google searches concerning the automotive market, holidays, job market conditions and teleworking (in local language) from `https://trends.google.com`.
Mobility (phone)	daily	2020-01	Mobility indicators based on mobile phone data from Santamaria et al. (2020)
News	daily	1995-01	Sentiment indicators about the economy, financial sector, manufacturing, inflation and monetary policy from Barbaglia et al. (2021)
Road tolls	daily	2008-01	Truck Toll Mileage Index for Germany, Calendar Adjusted from `www.destatis.de`
Total deaths	weekly	2000-01	Total deaths by Eurostat
Wikipedia	daily	2007-01	Wikipedia page views from `https://wikimedia.org/api/`

Table A.2: Independent variables: big data.

have mean zero and variance one. Here below we now provide detailed information on the transformation applied to the input data by each model.

For the ARDL unrestricted equations we adopt several transformations for each big data variable. We consider each transformation of a variable as an additional regressor, therefore in different equations the same variable may enter in levels, quarter-on quarter or month-on-month growth rates, and with different lags (up to three months). Non-stationary specifications are dropped. Estimation samples may therefore vary, depending on variable and transformation. Ragged edge issues are dealt with by using different lags, and dropping those models where the transformed indicator is not available.

The DFM picks up, for each country in the analysis, only 20 variables at monthly frequency. More precisely, the model contains: six text-based sentiment indicators by Barbaglia et al. (2021) related to the overall state of the economy, financial sector, industrial production, inflation and monopoly (these are daily variables that are aggregated monthly by averaging); six indicators produced by the Eurostat such as construction, consumer, industrial, retail and service confidence indicators and an economic sentiment indicator; five surveys from the European Commission which are the composite PMI output index, the construction PMI total activity index, manufacturing PMI new orders index, manufacturing PMI index, services PMI business activity index; two confidence indicators from OECD namely the consumers opinion surveys and the business tendency surveys (manufacturing). These variables enter in the model without any transformation since they are stationary by construction (see European Union, 2006 and the appendix of Giannone et al., 2009 for more details). For all the variables we consider a sample period which starts in 2000 and goes till the last monthly available observation. We deal with the ragged edge issue by imputing the last available data to missing observations. In the estimation procedure, no missing values are allowed.

The MG-MIDAS, thanks to the GLSS step, is able to use all the available monthly variables that have no missing observations. Variables are stationarized by differencing if an Augmented Dickey-Fuller test is accepted. This conservative procedure could result in overdifferentiation because we are not considering any correction for multiple testing (e.g., Bonferroni), but the model inference remains valid. The lags considered for the MIDAS weights are selected estimating different models for different set of lags and then selecting using Bayesian Information Criteria. Different variables could enter in the different lagged models according to their missing value structure. This takes also into consideration the ragged edge issue.

The MF-BVAR considers only a limited subset of the available variables, namely GDP, unemployment rate, CPI, the business and consumer confidence indicators, the PMI activity indicator and text-based sentiment measure about the overall state of the economy. Following Schorfheide and Song (2015), the variables enter the model in log levels, with the exception of the unemployment rate which is not log-transformed. The series that are available at weekly or daily frequency are monthly aggregated by averaging. In this way, variables that enter the MF-BVAR are either quarterly or monthly. We select the input variables such that no missing values are present at the begging of the sample. As it regards the presence of ragged edges, we fill the monthly series with missing data as in Ankargren and Jonéus (2021).

The ML models consider the full data set as input. The variables are aggregated at quarterly frequency by averaging and taken as percentage returns (e.g., the target variable GDP is log-transformed and taken in first difference). We expand the cross-section of the

input data by adding a one-quarter lag observation for each variable. As it regards the presence of incomplete data structures, each ML model deals with missing values in a different way[4]. For instance, NN deals with missing entries by mean imputation, while RF and XGB consider missing values as separate categorical labels.

Appendix B. Model predictive likelihood

Figure B.1 reports the predictive likelihood attached to each model listed in the main paper, namely autoregressive distributed lag models (ARDL), mixed-data sampling regressions (MIDAS), mixed-frequency Bayesian vector autoregression (MF-BVAR) and dynamic factors models (DFM), with some machine learning (ML) forecasting models. At each point in time, the predictive likelihood of each model is measured and reported (after normalization), so that a bar twice as big as another also reflects a predictive likelihood ratios of two among model types. The models' weights seems to be relatively stable before the pandemic, while the COVID-19 crisis imposes a different weighting scheme. On the one hand, in 2020 we observe higher weights attached to the ML and DFM models, which include big data variables as additional regressors and are able to more easily fit the non-linear shock imposed by the pandemic on the national economies. On the other hand, the weights attached to the MF-BVAR, MIDAS and the BMA equations are relatively smaller in 2020: while these models are dominant in normal times, their performance is drastically reduced with the pandemic breakthrough.

[4] We refer to `http://docs.h2o.ai/h2o/latest-stable/h2o-docs/automl.html` for the detail on how each ML models deal with missing values.

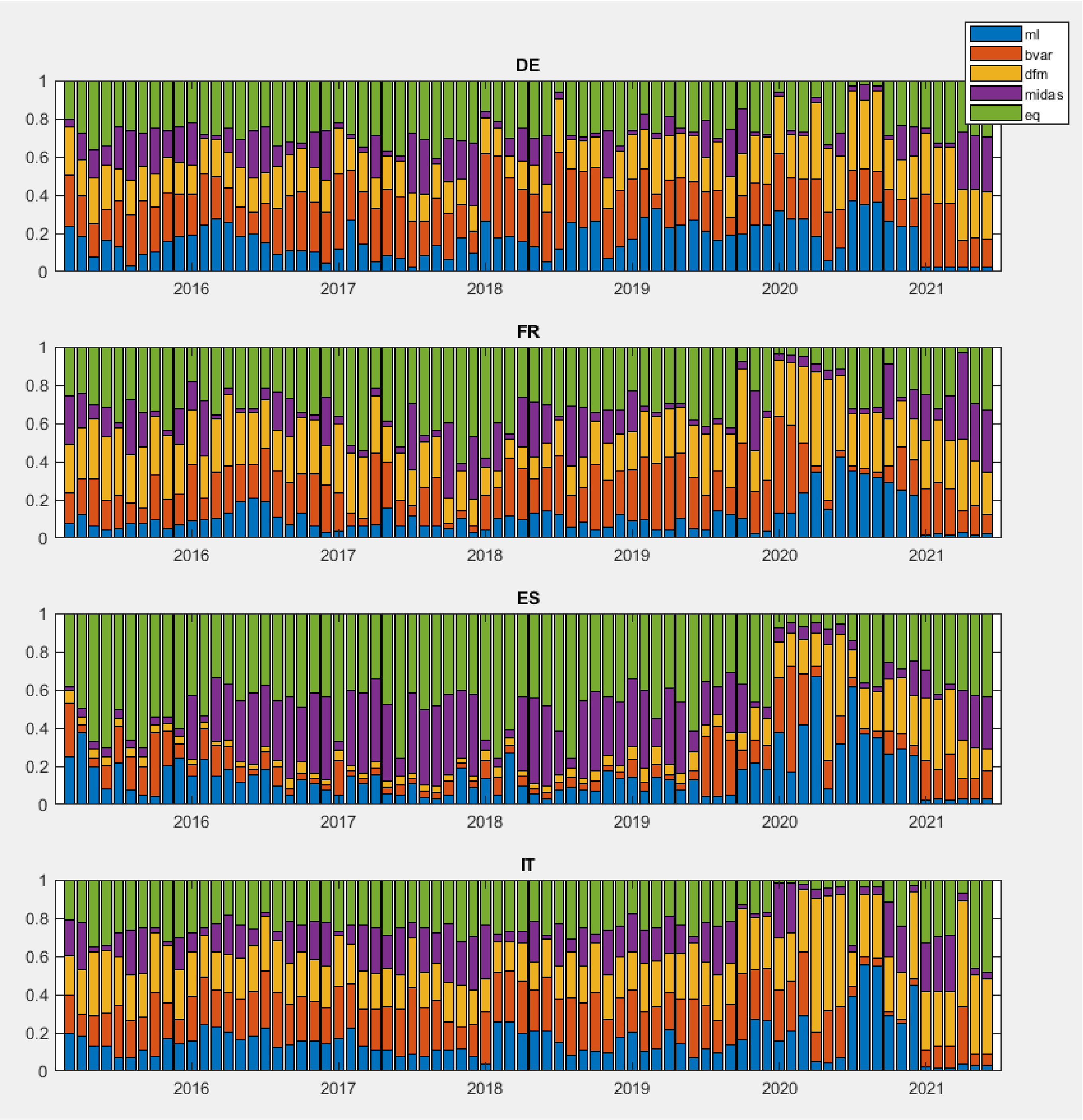

Figure B.1: Normalized predictive likelihood of each class of models (*ml*: Machine Learning, *bvar*: Mixed-Frequency Bayesian Vector AutoRegression, *midas*: Mixed Data Sampling, *dfm*: Dynamic Factor Model, *eq*: Bayesian Model Averaging unrestricted equations) at monthly frequency.

References

Ankargren S and Jonéus P (2021) Simulation smoothing for nowcasting with large mixed-frequency VARs. *Econometrics and Statistics* 19, 97–113

Barbaglia L, Consoli S and Manzan S (2021) Forecasting GDP in Europe with textual data. *Available at SSRN 3898680*

Consoli S, Pezzoli LT and Tosetti E (2021) Emotions in macroeconomic news and their impact on the European bond market. *Journal of International Money and Finance* 118, 102472

Dodds PS, Clark EM, Desu S, Frank MR, Reagan AJ, Williams JR, Mitchell L, Harris KD, Kloumann IM, Bagrow JP, Megerdoomian K, McMahon MT, Tivnan BF and Danforth CM (2014) Human language reveals a universal positivity bias. *Plos One* 66

European Union (2006) The Joint Harmonized EU Programme of Business and Economic Surveys. Technical report, Directorate-General for Economic and Fiancial Affairs - European Commission

Giannone D, Richelin L and Simonelli S (2009) Nowcasting Euro Area Economic Activity in Real Time: the Role of Confidence Indicators. *National Institute Economic Review* (210), 90–97

Iacus SM, Natale F, Santamaria C, Spyratos S and Vespe M (2020) Estimating and projecting air passenger traffic during the COVID-19 coronavirus outbreak and its socio-economic impact. *Safety Science* 129, 104791

Leetaru K and Schrodt PA (2013) Gdelt: Global data on events, location, and tone, 1979–2012. In *ISA annual convention*, volume 2. Citeseer, 1–49

Loughran T and McDonald B (2011) When is a liability not a liability? Textual analysis, dictionaries and 10-ks. *Journal of Finance* 66(1), 35–65

Martindale C (1987) Narrative Pattern Analysis: A Quantitative Method for Inferring the Symbolic Meaning of Narratives. In *Literary Discourse: Aspects of Cognitive and Social Psychological Approaches, Halasz L. (ed)*. De Gruyter, 167–181

Santamaria C, Sermi F, Spyratos S, Iacus SM, Annunziato A, Tarchi D and Vespe M (2020) Measuring the impact of COVID-19 confinement measures on human mobility using mobile positioning data. A European regional analysis. *Safety Science* 132, 104925

Schorfheide F and Song D (2015) Real-time forecasting with a mixed-frequency VAR. *Journal of Business & Economic Statistics* 33(3), 366–380

Schumacher C (2016) A comparison of MIDAS and bridge equations. *International Journal of Forecasting* 32(2), 257–270

Strapparava C and Valitutti A (2004) WordNet-Affect: An Affective Extension of WordNet. *Proceedings of the 4th International Conference on Language Resources and Evaluation (LREC 2004)* , 1083–1086

Wallis KF (1986) Forecasting with an econometric model: The ‘ragged edge’problem. *Journal of Forecasting* 5(1), 1–13
